# Overexpression of angiopoietin-1 reduces doxorubicin-induced apoptosis in cardiomyocytes

**DOI:** 10.7555/JBR.26.20120006

**Published:** 2012-06-06

**Authors:** Danyang Ren, Quan Zhu, Jiantao Li, Tuanzhu Ha, Xiaohui Wang, Yuehua Li

**Affiliations:** a Department of Pathophysiology, Nanjing Medical University, Nanjing, Jiangsu 210029, China;; b Department of Thoracic & Cardiovascular Surgery, the First Affiliated Hospital, Nanjing Medical University, Nanjing, Jiangsu 210029, China;; c Department of Surgery, James H. Quillen College of Medicine, East Tennessee State University, Johnson City, TN 37614, USA.

**Keywords:** cardiomyocyte, doxorubicin, apoptosis, angiopoietin-1, phosphoinositide-3 kinase (PI3K), nuclear factor-kappaB (NF-κB)

## Abstract

Doxorubicin (Dox) is a major anticancer chemotherapeutic agent. However, it causes cardiomyopathy due to the side effect of cardiomyocyte apoptosis. We have previously reported that angiopoietin-1 significantly reduced myocardial infarction after ischemic injury and protected cardiomyocytes from oxidative stress-induced apoptosis. It is hypothesized that angiopoietin-1 may protect cardiomyocytes from Dox-induced apoptosis. Cardiomyocytes H9C2 were transfected with adenovirus expressing angiopoietin-1 (Ad5-Ang-1) 24 h before the cells were challenged with Dox at a concentration of 2 µmol/L. Ad5-GFP served as the vector control. Cardiomyocyte apoptosis was evaluated using Annexin V-FITC staining and caspase-3 and caspase-8 activity was determined by Western blotting. The results showed that Dox treatment significantly induced cardiomyocyte apoptosis as evidenced by the greater number of Annexin V-FITC stained cells and increases in caspase-3 and caspase-8 activity. In contrast, overexpression of angiopoietin-1 significantly prevented Dox-induced cardiomyocyte apoptosis. To elucidate the mechanisms by which angiopoietin-1 protected cells from Dox-induced apoptosis, we analyzed both extrinsic and intrinsic apoptotic signaling pathways. We observed that angiopoietin-1 prevented Dox-induced activation of both extrinsic and intrinsic apoptotic signaling pathways. Specifically, angiopoietin-1 prevented DOX-induced increases in FasL and Bax levels and cleaved caspase-3 and caspase-8 levels in H9C2 cells. In addition, overexpression of angiopoietin-1 also activated the pro-survival phosphoinositide-3 kinase (PI3K)/Akt signaling pathway and decreased Dox-induced nuclear factor-kappaB (NF-κB) activation. Our data suggest that promoting the expression of angiopoietin-1 could be a potential approach for reducing Dox-induced cardiomyocyte cytoxicity.

## INTRODUCTION

Doxorubicin (Dox) is one of the most potent and effective chemotherapeutic agents for the treatment of various types of cancers[1]. However, the dose-dependent chronic cardiac toxicity of Dox limits its clinical application and may ultimately lead to cardiomyopathy and heart failure[2]. It has been reported that reactive oxygen species generation and myocardial apoptosis may be responsible for the pathogenesis of Dox-induced cardiac toxicity[3],[4]. At present, the exact mechanisms of Dox-induced cardiac toxicity remain unclear. Therefore, the search for effective and safe agents that will reduce Dox-induced cardiac toxicity may provide a new approach for promoting Dox in clinical applications.

Angiopoietin-1 (Ang-1) is a growth factor protein, which plays an important role in vascular development and angiogenesis[5]. Recent studies have shown that Ang-1 significantly promoted cardiac survival after myocardial ischemic injury and attenuated cell apoptosis[6]. It has been reported that Ang-1 inhibits Dox-induced human umbilical vein endothelial cell death by modulating Fas expression *via* the phosphoinositide-3 kinase (PI3K)/Akt pathway[7]. However, the role of Ang-1 in Dox-induced myocardial apoptosis has not been investigated. This study was designed to determine the protective effect of Ang-1 on Dox-induced apoptosis in cardiomyocytes. We observed that Ang-1 overexpression significantly attenuated Dox-induced apoptosis in H9C2 cardiomyocytes. The mechanisms involved blunting Dox-activated Fas-mediated apoptotic signaling pathway and activation of the pro-survival Akt signaling pathway.

## METHODS AND MATERIALS

### Cell culture and reagents

We employed rat embryonic heart-derived myoblast cell line H9C2 (American Type Culture Collection). The cells were maintained in RPMI-1640 medium (Mediatech, Washington, DC, USA) supplemented with 10% newborn calf serum (HyClone, Logan, UT, USA) in a humidified atmosphere containing 5% CO_2_ at 37°C. Dox and LY294002 were purchased from Sigma (St. Louis, MO, USA) and FITC-conjugated Annexin V from BD Biosciences (Mountain View, CA, USA).

### Cell treatment and gene transfection

Replication-deficient adenoviruses encoding human Ang-1 (Ad5-Ang1) were generated by homologous recombination as described previously[8]. Gene expression was driven by a cytomegalovirus promoter/enhancer. Ad5-green fluorescence protein (GFP) was used as a parallel control during gene transfection. Cardiomyocytes H9C2 were transfected with Ad5-Ang1 or Ad5-GFP at a multiplicity of infection (MOI; 20 plaque-forming units (PFUs)/cell). Twenty-four h after transfection, the cells were challenged with Dox at a concentration of 2 µmol/L. The cells were harvested for analysis of apoptosis and Western blot. There were three replicates in each group.

### Western blotting assays

Cytoplasmic proteins were prepared from harvested cells. Western blotting was performed as described previously[9]. Briefly, the cytoplasmic proteins were separated by sodium dodecyl sulfate polyacrylamide gel electrophoresis (SDS-PAGE) and transferred onto Hybond ECL nitrocellulose membranes (Amersham Pharmacia, San Francisco, CA, USA). The ECL membranes were incubated with the appropriate primary antibodies followed by incubation with peroxidase-conjugated secondary antibodies (Cell Signaling Technology Inc., Beverly, MA, USA). The signals were detected with the ECL system (Amersham Pharmacia). The same membranes were probed with antibody for glyceraldehyde-3-phosphate dehydrogenase (GAPDH; Biodesign International Inc., Kennebunk, Maine, USA), and then washed with stripping buffer. The signals were quantified by scanning densitometry and computer-assisted image analysis. The primary antibodies used in the study were anti-cleaved caspase-3, anti-cleaved caspase-8, anti-FasL, anti-Bax, anti-phospho-Akt (Ser473), and anti-Akt (t-Akt) antibodies (Cell signaling Technology, USA).

### Flow cytometric analysis

The assay was performed according to the manufacturer's instruction. Briefly, both treated and untreated cells were harvested, washed with PBS, suspended in Annexin V binding buffer (10 mmol/L HEPES, pH 7.4; 2.5 mmol/L CaCl_2_, 140 mmol/L NaCl), stained with Annexin V-FITC, and determined by flow cytometry using the FACScalibur flow cytometer (BD Biosciences, San Jose, CA, USA).

### Electrophoretic mobility shift assay (EMSA)

Nuclear proteins were isolated from H9C2 cells as described previously[9]. Nuclear factor-kappaB (NF-κB) binding activity was examined by EMSA in a 15 µL of binding reaction mixture containing 15 µg of nuclear proteins and 35 fmol of ^32^P-labeled double-stranded NF-κB consensus oligonucleotide. The signals were quantified by scanning densitometry and computer-assisted image analysis. The results were expressed as the ratio of the integrated density volume (IDV) of NF-κB to the average IDV of background.

### Statistical analysis

All results were expressed as mean±standard deviation (SD). For testing the differences of statistical significance between groups, one-way analysis of variance (ANOVA) and Student-Newnan-Keuls test were performed. A *P*-value < 0.05 was considered statistically significant.

## RESULTS

### Overexpression of Ang-1 suppresses DOX-induced apoptosis of cardiomyocytes

**Fig. 1:**
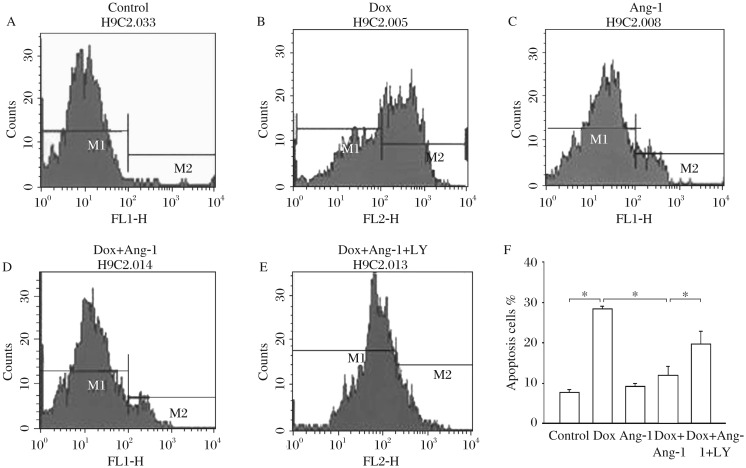
Overexpression of angiopoietin-1 (Ang-1) in cardiomyocytes attenuates doxorubicin (Dox)-induced apoptosis. H9C2 cells were transfected with adenovirus expressing Ang-1 (Ad5-Ang-1) and Ad5-GFP 24 h before the cells were treated with 2 µmol/L Dox. Untransfected H9C2 cells were treated with Dox (2 µmol/L) for 24 h. Percentage of apoptotic populations is represented as the M2-gated population. A: control group; B: Dox; C: Ang-1; D: Dox and Ang-1; E: Dox, Ang-1 and LY. LY: LY294002. *P < 0.05, *n* = 3/group.

To investigate the effect of overexpression of Ang-1 on DOX-induced apoptosis in cardiomyocytes, we employed flow cytometry to detect apoptotic cells stained with Annexin V-FITC. ***Fig. 1*** showed that Dox treatment significantly increased the proportion of apoptotic H9C2 cells (i.e., Annexin V positive staining cells) by 271% compared with the untreated cells. Consistently, Ad5-Ang-1 transfection significantly attenuated DOX-induced apoptosis by 59%. LY294002, a specific inhibitor of PI3-Kinase, abolished Ang-1-induced increase of apoptosis in H9C2 cardiomyocytes. There was no significant difference in the rate of apoptosis between the Ad5-Ang-1 and control groups.

### Overexpression of Ang-1 attenuated DOX-induced caspase-3 activity in cardiomyocytes

**Fig. 2:**
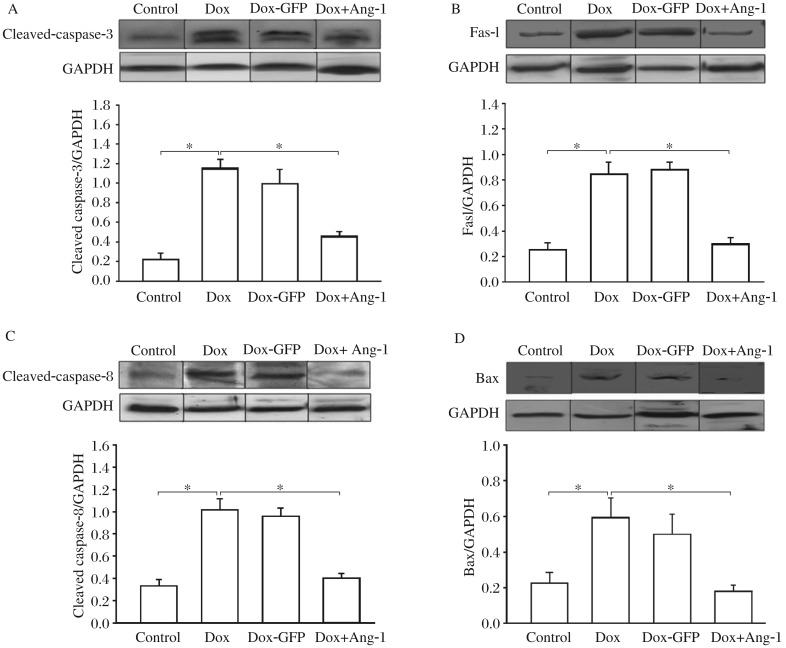
Overexpression of angiopoietin-1 (Ang-1) on doxorubicin (DOX)-induced caspase-3, FasL, caspase-8, and Bax in cardiomyocytes. H9C2 cells were transfected with adenovirus expressing Ad5-Ang-1 and Ad5-GFP 24 h before the cells were treated with 2 µmol/L Dox. Untransfected H9C2 cells were treated with Dox (2 µmol/L) for 24 h. Proteins were extracted for Western blot analysis with specific antibodies. A: overexpression of Ang-1 prevents DOX-induced caspase-3 activity. B: overexpression of Ang-1 attenuats Dox-induced increase in FasL level. C: overexpression of Ang-1 attenuats Dox-induced increase in cleaved caspase-8 level. D: overexpression of Ang-1 attenuated Dox-increased Bax. *P < 0.05. *n* = 3/group.

Increased caspase-3 activity is an established marker for cell apoptosis[10]. We examined caspase-3 activity in Dox-treated H9C2 cells in the presence or absence of Ang-1. As shown in ***Fig. 2A***, Dox treatment significantly increased cleaved caspase-3 level by 380% compared with the control cells. However, overexpression of Ang-1 significantly attenuated Dox-activated increase in cleaved caspase-3 level by 55.5%. Transfection of cells with Ad5-GFP did not affect Dox-induced increases in cleaved caspase-3 level.

### Overexpression of Ang-1 attenuated Dox-increased FasL and cleaved caspase-8 levels in cardiomyocytes

Fas is a cell surface receptor that recognizes Fas ligand (FasL), leading to the activation of caspase-8 mediated apoptotic signaling[11]. We examined the effect of Ang-1 overexpression on Dox-induced activation of FasL and its downstream caspase-8 activity in H9C2 cells. ***Fig. 2B*** showed that Dox treatment significantly increased FasL level by 230% compared with the untreated cells. In addition, Dox treatment significantly increased cleaved caspase-8 level by 207% compared with the untreated cells (***Fig. 2C***). Identically, transfection of the cells with Ad5-Ang-1 significantly prevented Dox-activated increases in FasL and caspase-8 levels. Transfection of the cells with Ad5-GFP did not affect Dox-activated increases in FasL and cleaved caspase-8 levels. These data suggested that Dox-induced apoptosis of H9C2 cells was mediated, in part, by activation of Fas-mediated apoptotic signaling pathway. Overexpression of Ang-1 significantly prevented Dox-activated increases in FasL and cleaved caspase-8 levels.

**Fig. 3:**
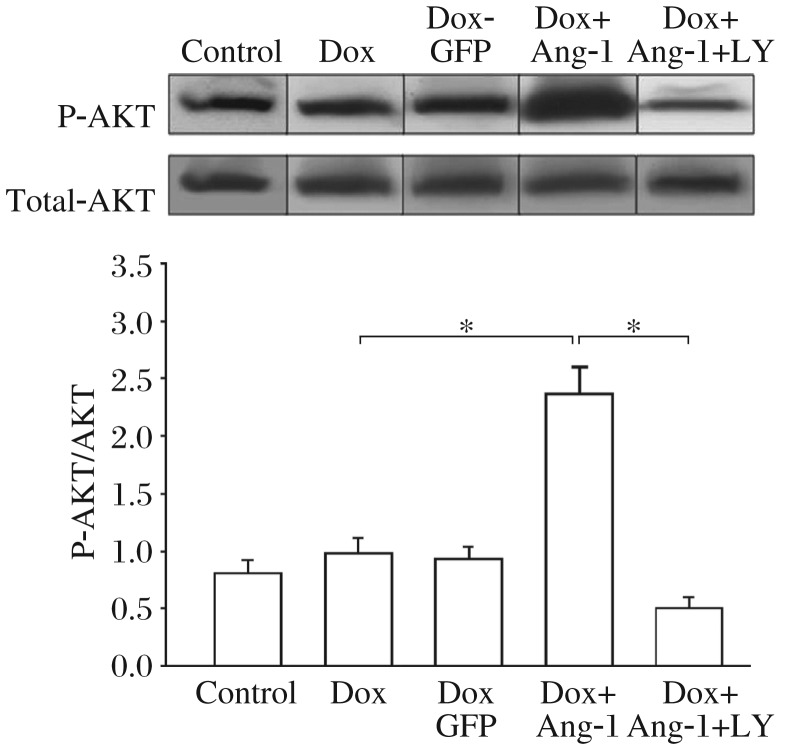
Overexpression of angiopoietin-1 (Ang-1) increases the levels of phosphorylated Akt (p-Akt) in doxorubicin (Dox)-treated cardiomyocytes. H9C2 cells were transfected with adenovirus expressing Ad5-Ang-1, Ad5-GFP 24 h before the cells were treated with 2 µM Dox. Untransfected H9C2 cells were treated with Dox (2 µM) for 24 h. Proteins were extracted for Western blot analysis with a specific antibody against p-Akt (Ser437) and total Akt. LY, LY294002. *P < 0.05, compared with indicated groups. *n* = 3/group.

### Overexpression of Ang-1 attenuated Dox-induced abundance of Bax in cardiomyocytes

We also examined the effect of Ang-1 on mitochondria-dependent apoptotic signaling pathway in Dox-treated H9C2 cells. In mitochondria-dependent apoptotic signaling pathway, cytochrome c, which is released by the mitochondria in response to pro-apoptotic stimuli, activates caspase-9 followed by activation of caspase-3, resulting in apoptosis. Bax is a pro-apoptotic protein, which can induce the release of cytochrome c from mitochondria[12]. Therefore, we examined the levels of Bax in Dox-treated H9C2 cells in the presence or absence of Ad5-Ang-1. ***Fig. 2D*** showed that Bax level was significantly increased by 159% compared with the untreated cells. Overexpression of Ang-1 significantly prevented Dox-induced increase in Bax level. Ad5-GFP transfection did not affect Dox-induced increase in Bax level in H9C2 cells. The data suggested that prevention of Dox-induced activation of mitochondria-mediated apoptotic signaling pathway could be one of the mechanisms by which Ang-1 protected cells against Dox-induced apoptosis.

### Overexpression of Ang-1 upregulated PI3K/AKT activity in DOX-treated H9C2 cardiomyocytes

It has been shown that activation of the PI3K/Akt signaling pathway plays an important role in regulating cell survival[13]. Ang-1 is a growth factor which activates the PI3K/Akt signaling pathway[14]. We examined the role of Ang-1 in activating the PI3K/Akt signaling pathway in Dox-treated H9C2 cells. ***Fig. 3*** showed that Dox treatment alone did not alter phospho-Akt level in H9C2 cells. However, overexpression of Ang1 significantly increased the level of phospho-Akt by 140% in Dox-treated cells compared with Dox-treated cells without Ang-1. Transfection of Ad5-GFP did not alter Akt level in Dox-treated cells. To determine whether increased Akt phosphorylation by Ang-1 is involved in the activation of PI3K, we employed LY294002, a specific inhibitor for PI3K in Ad5-Ang-1 expressed cells. As shown in ***Fig. 3***, LY294002 administration abolished Ang-1-induced increases in phospho-Akt level in H9C2 cardiomyocytes. The data suggested that Ang-1 upregulated the pro-survival PI3K/Akt signaling pathway, which could be an additional mechanism of Ang-1 protection against Dox-induced apoptosis.

### Overexpression of Ang-1 prevented Dox-induced increase in NF-κB binding activity in H9C2 cardiomyocytes

**Fig. 4:**
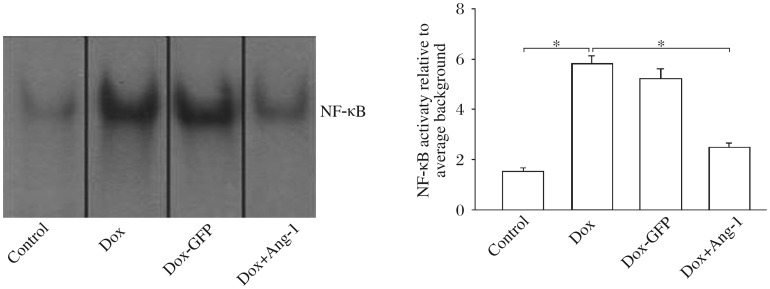
Overexpression of angiopoietin-1 (Ang-1) decreases the NF-κB binding activity in doxorubicin (Dox)-treated cardiomyocytes. H9C2 cells were transfected with adenovirus expressing Ad5-Ang-1 and Ad5-GFP 24 h before the cells were treated with 2 µmol/L Dox. Untransfected H9C2 cells were treated with Dox (2 µmol/L) for 24 h. The nuclear proteins were extracted for EMSA. *P < 0.05. *n* = 3/group.

NF-κB plays a key role in Dox-induced apoptosis[15]. Therefore, we investigated the effect of Ang-1 overexpression on Dox-induced NF-κB activation. As shown in ***Fig. 4***, NF-κB binding activity was significantly increased by 228% in Dox-treated cells compared with the untreated cells. Overexpression of Ang-1 significantly prevented Dox-induced increase in NF-κB binding activity (***Fig. 4***). Transfection of the cells with Ad5-GFP did not affect Dox-induced increases in NF-κB binding activity.

## DISCUSSION

In this study, we aimed to determine the effect of Ang-1 overexpression on Dox-induced apoptosis in H9C2 cardiomyocytes. We observed that overexpression of Ang-1 protected H9C2 cells from Dox-induced apoptosis. The mechanisms involved attenuation of Dox-induced both Fas-mediated and mitochondria-mediated apoptotic signaling pathways. In addition, overexpression of Ang-1 activated the pro-survival PI3K/Akt signaling pathway and decreased Dox-induced NF-κB activation. Our data suggest that promoting expression of Ang-1 could be a potential approach for reducing Dox-induced cardiomyocyte cytotoxicity.

Ang-1 is one type of angiopoietins, which are protein growth factors promoting angiogenesis, the formation of blood vessels from pre-existing blood vessels[16]. Ang-1 binds to its receptor Tie-2, a receptor tyrosine kinase expressed primarily on vascular endothelial cells[17]. Ang-1/Tie-2 signaling promotes angiogenesis during the development, remodeling, and repair of the vascular system[18]. Recent studies have shown that Ang-1 can improve cardiac function after myocardial ischemic injury[19] and sepsis and septic shock[20]. It has been previously reported that overexpression of Ang-1 significantly reduces myocardial infarction after myocardial ischemia injury and protects cardiac myocytes against oxidative stress-induced apoptosis[21]. Taken together, it is hypothesized that Ang-1 would protect cardiomyocytes against Dox-induced apoptosis.

Dox has been used for chemotherapy of cancer for at least three decades. However, its clinical application is limited because of its dose-dependent and progressive cardiomyopathy[22]. Cardiomyocyte apoptosis plays a critical role in Dox-induced cardiomyopathy[23]. The present study observed that Dox-induced apoptosis of H9C2 cardiomyocytes via activation of both Fas-mediated (extrinsic) and mitochondria-mediated (intrinsic) apoptotic signaling pathways. Indeed, it has been well documented that Dox-induced cardiomyocyte apoptosis involves activation of both extrinsic and intrinsic apoptotic signaling pathways[24]. In extrinsic apoptotic signaling pathway, the cell surface receptor Fas recruits a cytosolic FAS-associated death domain (FADD) protein after FasL stimulation. Thus, the Fas, FasL and FADD become a cytosolic complex, which subsequently activates caspase-8[25]. In the intrinsic apoptotic signaling pathway, damaged mitochondria release cytochrome c, a 13-kDa heme-containing protein, leading to caspase-9 activation via the formation of the apoptosome complex containing cytochrome c, pro-caspase-9 and apoptosis activating factor (Apaf-1)[26]. Both apoptotic signaling pathways finally activate common effector caspase-3 that executes the apoptosis process. Importantly, overexpression of Ang-1 by transfection of Ad5-Ang-1 into H9C2 cells significantly prevented Dox-induced activation of both extrinsic and intrinsic apoptotic signaling pathway. Thus, promotion of Ang-1 expression could be a potential approach for treating Dox-induced cardiomyopathy.

It has been demonstrated that activation of the PI3K/Akt signaling pathway plays an important role in regulating of cell growth and survival[27]. Ang-1/Tie-2 activates downstream signaling pathways, including PI3K/Akt signaling. In the present study, we observed that overexpression of Ang-1 significantly increased phosphorylated Akt level with the presence of Dox. The data suggest that activation of the PI3K/Akt signaling pathway may be responsible for the protection against Dox-induced apoptosis. To evaluate this hypothesis, we administered LY294002, a specific PI3K inhibitor, to the cells, and observed that Ang-1-induced protection against Dox was abrogated. Thus, it is concluded that overexpression of Ang-1 protects H9C2 cells against Dox-induced apoptosis, which is mediated, in part, via activation of the PI3K/Akt signaling pathway.

Activation of NF-κB plays a critical role in the induction of immune and inflammatory responses[28]. NF-κB activation also regulates the expression of Fas, FasL, and p53, which are important mediators for apoptosis[29],[30]. We observed in the present study that Dox treatment significantly increased NF-κB binding activity. However, overexpression of Ang-1 prevented Dox-induced NF-κB binding activity in H9C2 cells. At present, the mechanism by which overexpression of Ang-1 prevents Dox-induced increase in NF-κB binding activity remains unclear. However, it has been shown that activation of the PI3K/Akt signaling pathway negatively regulates NF-κB activation[31].

In summary, we observed that overexpression of Ang-1 significantly attenuated Dox-induced apoptosis in H9C2 cells. Further studies will focus on *in*-*vivo* experiments to demonstrate the protective effect of Ang-1 on Dox-induced cardiomyopathy.
